# The Tohoku Medical Megabank Project: Design and Mission

**DOI:** 10.2188/jea.JE20150268

**Published:** 2016-09-05

**Authors:** Shinichi Kuriyama, Nobuo Yaegashi, Fuji Nagami, Tomohiko Arai, Yoshio Kawaguchi, Noriko Osumi, Masaki Sakaida, Yoichi Suzuki, Keiko Nakayama, Hiroaki Hashizume, Gen Tamiya, Hiroshi Kawame, Kichiya Suzuki, Atsushi Hozawa, Naoki Nakaya, Masahiro Kikuya, Hirohito Metoki, Ichiro Tsuji, Nobuo Fuse, Hideyasu Kiyomoto, Junichi Sugawara, Akito Tsuboi, Shinichi Egawa, Kiyoshi Ito, Koichi Chida, Tadashi Ishii, Hiroaki Tomita, Yasuyuki Taki, Naoko Minegishi, Naoto Ishii, Jun Yasuda, Kazuhiko Igarashi, Ritsuko Shimizu, Masao Nagasaki, Seizo Koshiba, Kengo Kinoshita, Soichi Ogishima, Takako Takai-Igarashi, Teiji Tominaga, Osamu Tanabe, Noriaki Ohuchi, Toru Shimosegawa, Shigeo Kure, Hiroshi Tanaka, Sadayoshi Ito, Jiro Hitomi, Kozo Tanno, Motoyuki Nakamura, Kuniaki Ogasawara, Seiichiro Kobayashi, Kiyomi Sakata, Mamoru Satoh, Atsushi Shimizu, Makoto Sasaki, Ryujin Endo, Kenji Sobue, the Tohoku Medical Megabank Project Study Group, Masayuki Yamamoto

**Affiliations:** 1 Tohoku Medical Megabank Organization, Tohoku University, Sendai, Japan; 1 東北大学 東北メディカル・メガバンク機構; 2 Graduate School of Medicine, Tohoku University, Sendai, Japan; 2 東北大学 大学院医学系研究科; 3 International Research Institute of Disaster Science, Tohoku University, Sendai, Japan; 3 東北大学 災害科学国際研究所; 4 Tohoku University Hospital, Tohoku University, Sendai, Japan; 4 東北大学病院; 5 Graduate School of Dentistry, Tohoku University, Sendai, Japan; 5 東北大学 大学院歯学研究科; 6 Institute of Development, Aging and Cancer, Tohoku University, Sendai, Japan; 6 東北大学 加齢医学研究所; 7 Graduate School of Information Sciences, Tohoku University, Sendai, Japan; 7 東北大学 大学院情報科学研究科; 8 Iwate Tohoku Medical Megabank Organization, Disaster Reconstruction Center, Iwate Medical University, Yahaba, Iwate, Japan; 8 岩手医科大学 いわて東北メディカル・メガバンク機構; 9 School of Medicine, Iwate Medical University, Morioka, Japan; 9 岩手医科大学 医学部; 10 Institute for Biomedical Science, Iwate Medical University, Yahaba, Iwate, Japan; 10 岩手医科大学 医歯薬総合研究所

**Keywords:** the Great East Japan Earthquake, Tohoku Medical Megabank Project, cohort study, biobank, genomic research, 東日本大震災, 東北メディカル・メガバンク計画, コホート調査, バイオバンク, ゲノム解析研究

## Abstract

The Great East Japan Earthquake (GEJE) and resulting tsunami of March 11, 2011 gave rise to devastating damage on the Pacific coast of the Tohoku region. The Tohoku Medical Megabank Project (TMM), which is being conducted by Tohoku University Tohoku Medical Megabank Organization (ToMMo) and Iwate Medical University Iwate Tohoku Medical Megabank Organization (IMM), has been launched to realize creative reconstruction and to solve medical problems in the aftermath of this disaster. We started two prospective cohort studies in Miyagi and Iwate Prefectures: a population-based adult cohort study, the TMM Community-Based Cohort Study (TMM CommCohort Study), which will recruit 80 000 participants, and a birth and three-generation cohort study, the TMM Birth and Three-Generation Cohort Study (TMM BirThree Cohort Study), which will recruit 70 000 participants, including fetuses and their parents, siblings, grandparents, and extended family members. The TMM CommCohort Study will recruit participants from 2013 to 2016 and follow them for at least 5 years. The TMM BirThree Cohort Study will recruit participants from 2013 to 2017 and follow them for at least 4 years. For children, the ToMMo Child Health Study, which adopted a cross-sectional design, was also started in November 2012 in Miyagi Prefecture. An integrated biobank will be constructed based on the two prospective cohort studies, and ToMMo and IMM will investigate the chronic medical impacts of the GEJE. The integrated biobank of TMM consists of health and clinical information, biospecimens, and genome and omics data. The biobank aims to establish a firm basis for personalized healthcare and medicine, mainly for diseases aggravated by the GEJE in the two prefectures. Biospecimens and related information in the biobank will be distributed to the research community. TMM itself will also undertake genomic and omics research. The aims of the genomic studies are: 1) to construct an integrated biobank; 2) to return genomic research results to the participants of the cohort studies, which will lead to the implementation of personalized healthcare and medicine in the affected areas in the near future; and 3) to contribute the development of personalized healthcare and medicine worldwide. Through the activities of TMM, we will clarify how to approach prolonged healthcare problems in areas damaged by large-scale disasters and how useful genomic information is for disease prevention.

## Abstract (ja)

2011年3月11日に発生した東日本大震災は、東北地方の太平洋沿岸に甚大な被害をもたらした。震災からの復興を促進し、災害後の医療上の問題を解決するために、東北大学は東北メディカル・メガバンク計画を立案し、岩手医科大学と協働して、宮城県と岩手県において、地域住民コホート調査及び三世代コホート調査と名付けた2つの前向きコホート調査を開始した。地域住民コホート調査では、被災地を中心に80,000人の成人をリクルートする、また、三世代コホート調査では、妊婦と配偶者、出生した子供、その兄姉、祖父母あわせて70,000人をリクルートする予定である。前者では、2013年から2016年まで参加者のリクルートを実施し、その後少なくとも5年間追跡する、また、後者では、2013年から2017年まで参加者をリクルートし、その後少なくとも4年間追跡する予定である。これらに加え、2012年から宮城県で横断調査デザインからなる地域子ども長期健康調査を開始している。東北メディカル・メガバンク計画では、これら2つの前向きコホート調査をもとに、生体試料とゲノム情報を含む関連情報から構成される複合バイオバンクを構築する。本複合バイオバンクには、健康と医療に関する情報、生体試料、ゲノムデータ等が保管され、主として東日本大震災によって増加や症状の悪化等が懸念される疾患について個別化予防・医療を実現するための基盤を確立することを目指している。なお、生体試料や情報は、外部の研究者へも分譲する予定である。本計画では、これらの活動を通して東日本大震災の住民の健康への中長期的な影響を調査する。東北メディカル・メガバンク計画では、コホート調査、バイオバンク形成とともに、ゲノム解析研究を推進する。その目的は、１）複合バイオバンクを構築する、２）コホート調査の参加者へゲノム解析結果を回付し、被災地において個別化予防・医療を推進する、３）人類全体に対して、個別化予防・医療の発展に貢献する、ことである。東北メディカル・メガバンク計画の活動を通して、大規模災害で被害を受けた地域における医療問題の解決方法及び疾病予防のためのゲノム情報の有益さを明らかにすることを目指す。

*東北メディカル・メガバンク計画スタディ・グループの名簿はAppendix を参照

## INTRODUCTION

**Figure 1.**
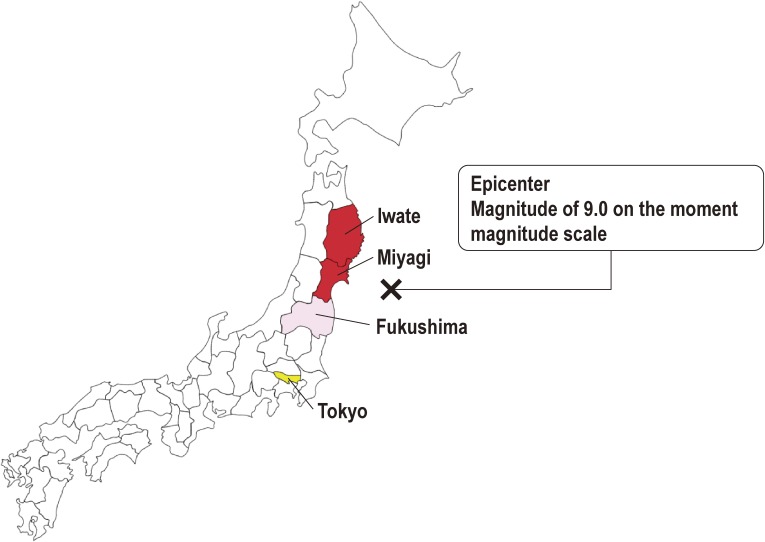
Location of Iwate Prefecture, Miyagi Prefecture, and Fukushima Prefecture and characteristics of the Great East Japan Earthquake This panel shows a map of Japan highlighting the location of Miyagi and Iwate Prefectures in red. Fukushima Prefecture, shown in light red color, is not included in this project. These three prefectures are areas most affected by the earthquake. Tokyo is shown in yellow color. The letter X indicates the epicenter.

The Great East Japan Earthquake (GEJE) of March 11, 2011 caused profound damage in wide areas of the Pacific coast of the Tohoku region of Japan.^1^ The tsunami hit the northeastern part of Honshu Island, and Iwate, Miyagi, and Fukushima Prefectures were severely damaged (Figure 1). In total, 15 827 people were lost and 2559 remain missing in these three prefectures.^2^ As of December 10, 2015, 182 000 people were still evacuees throughout Japan.^3^ Nearly 80% of the hospitals in Iwate, Miyagi, and Fukushima Prefectures were damaged,^4^ which severely affected the delivery of medical services. A detailed report of hospitals’ damage by the GEJE is available for Miyagi Prefecture.^5^

Since the GEJE, many studies have reported that the survivors of the disaster are suffering various set-backs, including unbalanced nutrition; lack of appropriate medical follow-ups; and psychological stresses due to uncomfortable living conditions and the loss of family members, relatives, friends, and colleagues, as well as homes and places of work.^6^ These factors accelerate the onset of or aggravate a variety of chronic, non-infectious diseases, such as peptic ulcer,^7^ ulcerative colitis,^8^ cardiovascular disorders,^9^^–^^16^ cerebrovascular disorders,^17^ diabetes mellitus,^18^^,^^19^ and chronic obstructive pulmonary disease.^20^ Preceding acute-phase studies on the epidemiological impacts of the GEJE revealed that the earthquake caused prolonged social, economic, and psychological suffering among the victims and their communities. Also, middle- or long-term impacts of natural disasters, such as the GEJE, on the health of affected persons are concerning.^21^^,^^22^ Moreover, convincing lines of evidence regarding the impacts of the GEJE on child health are still lacking, despite the presence of great concern.^23^

Maintaining sustainable medical practices in the tsunami-affected areas was challenging and complicated even before the GEJE occurred. As the tsunami-stricken areas have continuously suffered from an unbalanced distribution of physicians,^24^^–^^26^ simply reconstructing the damaged hospitals may not be sufficient to ensure stable restoration of medical services. Therefore, establishing an advanced medical system and practice is essential to the sustainable restoration of medical services in the tsunami-stricken areas. We surmise that personalized healthcare and medicine based on genomic information is one of the advanced medical practices that will support this purpose. Disease susceptibility and drug responsiveness are known to largely correlate with genetic factors, and personal genomic information can be an ultimate biomarker for many clinically important human traits.^27^

**Figure 2.**
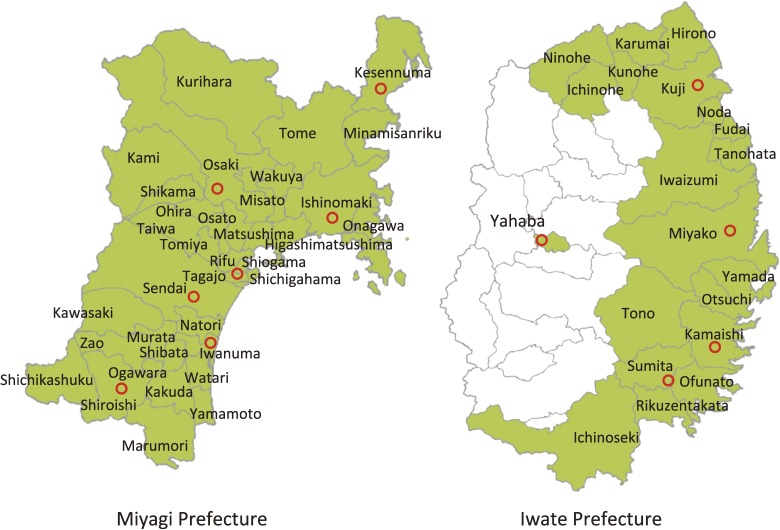
Municipalities of Miyagi Prefecture and Iwate Prefecture This panel shows the municipalities of Miyagi Prefecture and Iwate Prefecture. Green area indicates study area. Red circles indicate the location of the Community Support Centers in Miyagi Prefecture and the Satellites in Iwate Prefecture.

To this end, we established the Tohoku Medical Megabank Project (TMM), which is conducted by Tohoku University Tohoku Medical Megabank Organization (ToMMo) and Iwate Medical University Iwate Tohoku Medical Megabank Organization (IMM). We decided to conduct TMM in Miyagi and Iwate Prefectures (Figure 1 and Figure 2), since a number of similar large-scale projects have already been started in Fukushima Prefecture, including the Fukushima Health Management Survey,^28^ to promote the future well-being of their residents.

The mission of TMM is to facilitate solutions to medical problems in the aftermath of the GEJE by introducing personalized healthcare and medicine into the damaged areas. TMM has undertaken two prospective cohort studies in Miyagi and Iwate Prefectures. TMM also has contributed to the dispatch of young physicians to clinics and hospitals in the tsunami-suffered areas, and to the establishment of an integrated biobank equipped with an information-knowledge database that combines health and clinical information with data derived from genome analyses, such as whole-genome sequencing and custom-made single nucleotide polymorphism (SNP) arrays. Biospecimens are being collected and stored in a manner that facilitates the full range of ‘omics’ analyses. Through these activities, we aim to realize personalized healthcare and medicine based on genetic and lifestyle information from the residents in the earthquake- and tsunami-damaged areas. These activities will be helpful in recovery efforts and in improving these residents’ health status. Our additional aim is to contribute to the restoration and development of the damaged areas by undertaking various research activities that utilize biospecimens and information in the biobank and database. This paper outlines the mission, methodology, and progress of TMM.

## DESIGN

### Outline of the Tohoku Medical Megabank Project (TMM)

**Figure 3.**
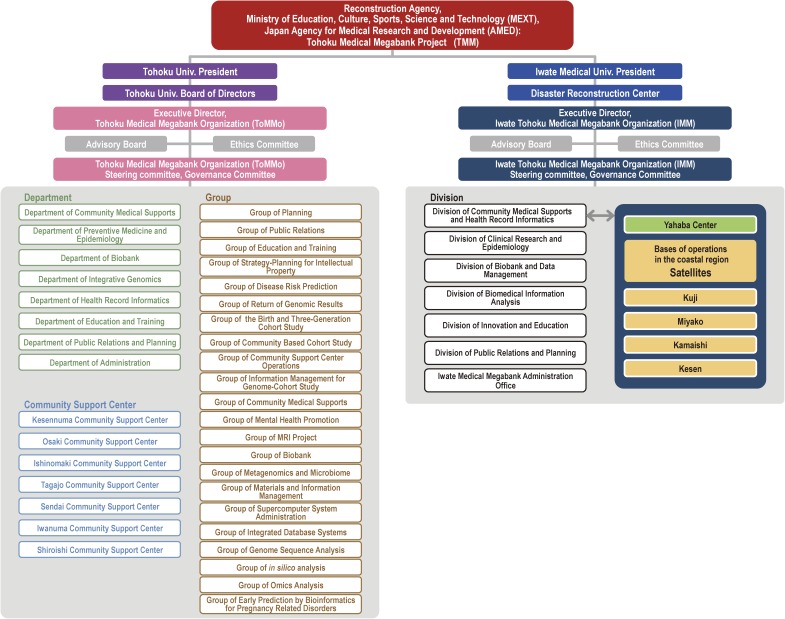
Organization of the Tohoku Medical Megabank Project (TMM) TMM started on February 1, 2012, and is sponsored by the Reconstruction Agency, the Ministry of Education, Culture, Sports, Science and Technology (MEXT), and the Japan Agency for Medical Research and Development (AMED). Panel shows the organization of TMM as of April 1, 2015.

TMM started on February 1, 2012, with the goals of assisting medical and health services to overcome the damages from the GEJE, treating diseases among the survivors, and implementing the most advanced medical services, including personalized healthcare and medicine, for current and future generations in the affected areas. We also hope to expand the scope of the project to contribute to health promotion not only for persons in the affected areas, but also for the whole human family. TMM is sponsored by the Reconstruction Agency; the Ministry of Education, Culture, Sports, Science and Technology (MEXT); and the Japan Agency for Medical Research and Development (AMED). ToMMo and IMM are responsible for the operation and development of TMM (Figure 3).

TMM mainly consists of three initiatives. First, we started two prospective cohort studies and one cross-sectional study. Second, we are constructing an integrated biobank that stores biospecimens and information collected through the cohort studies. Third, we perform regular genome and omics analyses with the collected biospecimens, and the resulting information will be stored in the biobank and distributed to the research community.

#### Cohort studies

The aims of TMM cohort studies are: 1) to monitor the effects of the damage caused by the GEJE on health status in the affected areas; 2) to contribute to the early diagnosis and treatment of potentially increasing diseases according to the results of the monitoring; and 3) to conduct molecular-epidemiological studies to clarify the associations between genetic factors, environmental factors, and diseases.

In order to conduct cohort studies in the affected areas, we first made efforts to recover the damaged healthcare services. In the affected areas, the shortage of physicians was critical even before the earthquake. To address this issue, we established a system to dispatch physicians to areas suffering from a physician shortage on a rotating basis. The system is called the “ToMMo Clinical Fellowship (TCF)” in Miyagi Prefecture and the “IMM Medical Megabank Fellows System” in Iwate Prefecture.

**Figure 4.**
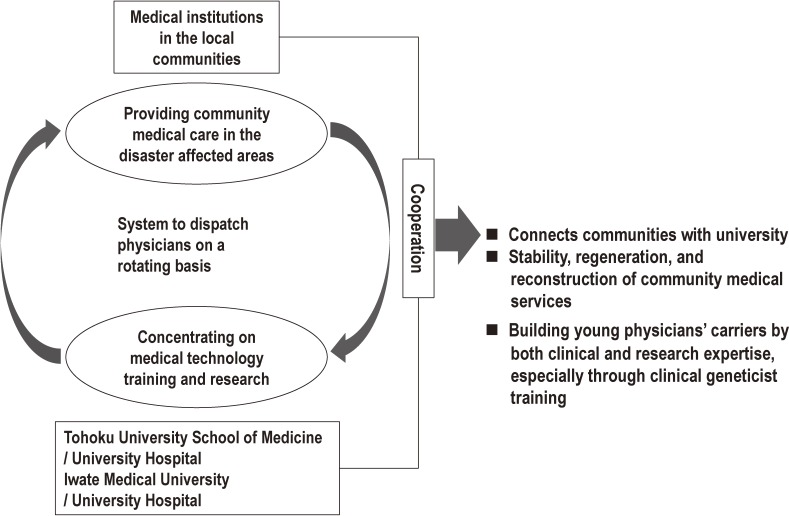
System of dispatching physicians on a rotating basis for community medical support and development of young resources ToMMo, jointly with Tohoku University Hospital and Tohoku University School of Medicine, has created the system of dispatching physicians on a rotating basis, which connects communities with the university. The system is referred to as the “ToMMo Clinical Fellowship (TCF)”. IMM has also adopted the same system, and refers to it as the “IMM Medical Megabank Fellows System”.

This physician dispatch system has many advantages. It offers advanced medical care to patients through the activities of young physicians involved in various specialties, and it aims to increase both the clinical and research expertise of young physicians, especially through clinical geneticist training (Figure 4). This physician dispatch system began on October 1, 2012. As of October 1, 2015, 40 physicians were dispatched to medical institutions in local communities, mainly along the Pacific coast. They have been working to support medical institutions in the affected areas, but they do not obtain consent from their patients or persons in the community to participate in the TMM cohort studies described below.

**Figure 5.**
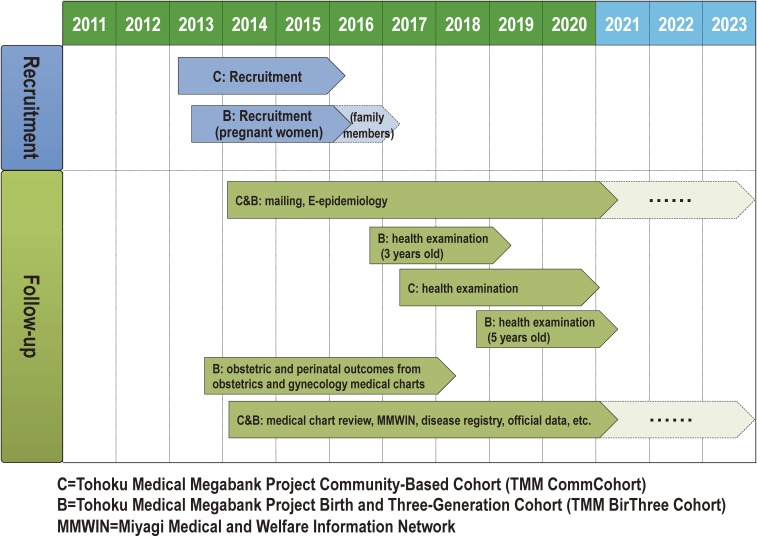
Roadmap of the TMM cohort studies This panel shows the roadmap for recruitment and follow-ups of the cohorts towards the realization of personalized healthcare and medicine.

In May 2013, we started a population-based adult cohort study named the TMM Community-Based Cohort Study (TMM CommCohort Study), which targets 80 000 participants: 50 000 from Miyagi Prefecture and 30 000 from Iwate Prefecture (Table 1 and Figure 5). The main target population of the TMM CommCohort Study is persons living in coastal areas in these two prefectures (Figure 2), but the study also includes persons living in inland areas.

**Table 1.** Characteristics of two cohort studies and one cross-sectional study in the Tohoku Medical Megabank Project (TMM)

	TMM Community-Based Cohort Study (TMM CommCohort Study)	TMM Birth and Three-Generation Cohort Study (TMM BirThree Cohort Study)	ToMMo Child Health Study
Study design	population and health checkup-based prospective cohort	population and hospital-based prospective cohort	school-based cross-sectional study

Recruitment or conduct period	May 2013 to March 2016	July 2013 to September 2016 for pregnant women	2012 to 2015
		July 2013 to March 2017 for family members	

Number of total expected participants			
Miyagi Prefecture	50 000 (recuruited mainly by ToMMo)	about 70 000	17 000
		(20 000 families)	
Iwate Prefecture	30 000 (recuruited mainly by IMM)	some	0

Ages	20 years old or older	fetus, 0 years old or older	school age, from 7 years old to 14 years old

Family information	partially available	almost fully available	none
	mainly partner’s information	three-generation and extended-family information	

Main target diseases	chronic diseases	pregnant and perinatal period diseases	allergic diseases
	mental diseases	allergic diseases	developmental disorders
	infectious diseases	developmental disorders	mental diseases
		chronic diseases	infectious diseases
		mental diseases	
		infectious diseases	

TMM, Tohoku Medical Megabank Project; ToMMo, Tohoku University Tohoku Medical Megabank Organization; IMM, Iwate Medical University Iwate Tohoku Medical Megabank Organization.

In the TMM CommCohort Study, we have been recruiting potential participants by two approaches. We recruit participants on the sites for specific health checkups of the annual community health examination, which are conducted by municipalities.^29^ The health checkups, which are conducted for insured persons aged from 40 to 74 years, focus on visceral fat obesity. Additionally, we have established facilities of seven “Community Support Centers” in Miyagi Prefecture and five “Satellites” in Iwate Prefecture for the voluntary admission-type recruitment and health assessment of participants (Figure 2).

**Figure 6.**
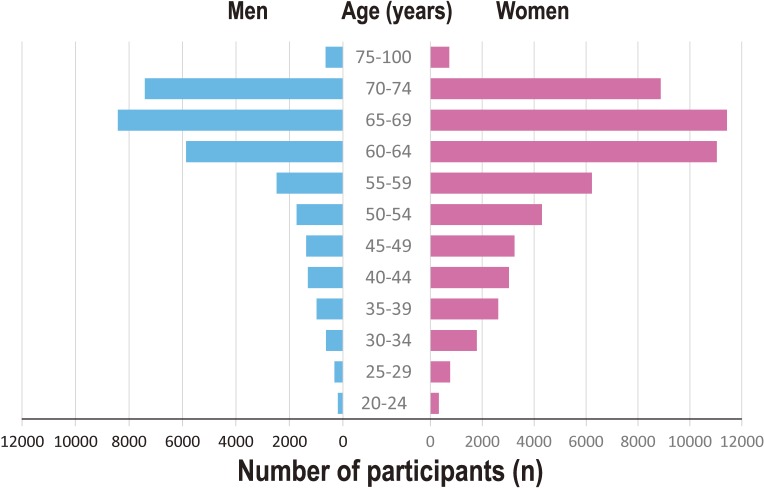
Age distribution of the TMM Community-Based Cohort (TMM CommCohort) participants This panel shows the age-distribution of participants in the TMM CommCohort as of December 28, 2015, recruited in Miyagi and Iwate Prefectures combined.

The inclusion criteria for the TMM CommCohort Study are: 1) for the specific health checkups sites based survey, persons aged 40 to 74 years who are registered in the basic resident register of all 35 municipalities in Miyagi Prefecture and 20 municipalities in Iwate Prefecture (Figure 2) at the time of enrollment; and 2) for the Community Support Center or the Satellite based survey, persons aged 20 years or over who are registered in the basic resident register of all 35 municipalities in Miyagi Prefecture and 20 municipalities in Iwate Prefecture (Figure 2) at the time of enrollment. The exclusion criteria for the TMM CommCohort Study are: 1) persons who do not consent to participate in the study; and 2) persons who are not able to fill out study questionnaires. As of December 28, 2015, 53 882 persons in Miyagi Prefecture and 31 838 persons in Iwate Prefecture have already participated in the TMM CommCohort Study (Table 2A and Table 2B). Figure 6 shows the age distribution of these participants. As of December 28, 2015, the participation response rates among health checkup examinees recruited on the sites for specific health checkups to the TMM CommCohort were 65% in Miyagi Prefecture and 77% in Iwate Prefecture.

**Table 2A.** Municipalities, population, and human damage by the GEJE^a^ and number of participants in the TMM CommCohort and the TMM BirThree Cohort Studies in Miyagi Prefecture

Community Support Center	Municipality	Population (as of October 1, 2015)	Human damage by the GEJE^a^	Number of participants (as of December 28, 2015)								

				TMM CommCohort				TMM BirThree Cohort				
	
				Specific health checkups	Community Support Center	Sub total	Total (*n* = 53 882)	Obstetric clinics and hospitals	Community Support Center	Others^b^	Sub total	Total (*n* = 44 309)
Kesennuma	Kesennuma	65 372	1326	721	1303	2024	2557	845	150	8	1003	1245
	Minamisanriku	13 482	812	518	15	533		215	25	2	242	

Osaki	Osaki	132 864	2	8504	947	9451	19 094	2722	166	53	2941	6982
	Kurihara	69 692	0	3589	148	3737		957	71	30	1058	
	Tome	80 677	4	2261	74	2335		1600	95	22	1717	
	Kami	23 841	0	982	51	1033		337	28	2	367	
	Shikama	7162	0	24	4	28		139	10	1	150	
	Wakuya	16 672	2	439	41	480		288	22	1	311	
	Misato	24 724	0	1903	127	2030		388	48	2	438	

Ishinomaki	Ishinomaki	145 760	3705	1402	1154	2556	5355	1995	347	22	2364	3170
	Higashimatsushima	39 759	1086	2333	196	2529		628	84	16	728	
	Onagawa	6631	850	249	21	270		67	11	0	78	

Tagajo	Shiogama	54 168	24	1311	462	1773	7901	531	112	8	651	2706
	Tagajo	62 314	188	2339	617	2956		1099	170	29	1298	
	Matsushima	14 499	2	972	59	1031		77	28	3	108	
	Shichigahama	18 709	78	894	98	992		150	34	4	188	
	Rifu	35 748	1	823	326	1149		363	90	8	461	

Sendai	Sendai	1 076 030	685	49	4519	4568	9775	17 367	1636	358	19 361	21 332
	Taiwa	28 056	1	1499	71	1570		620	29	22	671	
	Osato	8331	1	308	15	323		80	9	5	94	
	Tomiya	51 544	0	3136	154	3290		989	69	33	1091	
	Ohira	5687	0	17	7	24		105	10	0	115	

Iwanuma	Iwanuma	44 162	181	1242	552	1794	4437	1158	111	28	1297	4014
	Natori	77 041	951	663	482	1145		1431	152	29	1612	
	Watari	33 271	269	963	181	1144		758	86	9	853	
	Yamamoto	12 495	698	299	55	354		236	15	1	252	

Shiroishi	Shiroishi	35 084	0	1116	526	1642	4757	749	61	7	817	4292
	Kakuda	29 819	0	435	117	552		610	79	4	693	
	Zao	12 300	0	771	81	852		253	23	7	283	
	Shichikashuku	1453	0		3	3		8	2	0	10	
	Ogawara	23 699	0	129	184	313		656	52	4	712	
	Murata	11 361	0	503	47	550		294	20	8	322	
	Shibata	39 104	2	16	221	237		930	75	28	1033	
	Kawasaki	9129	0	1	23	24		96	4	1	101	
	Marumori	14 043	0	565	19	584		293	28	0	321	

Municipalities other than Miyagi Prefecture				4	2	6	6	354	189	25	568	568

GEJE, Great East Japan Earthquake; TMM, Tohoku Medical Megabank Project.

^a^Human damage by the GEJE includes dead and missing persons. The nubers are cited from the Miyagi Prefecture Crisis Measures division.

^b^Others include participants’ house, event site, etc.

**Table 2B.** Municipalities, population, and human damage from the GEJE^a^ and number of participants in the TMM CommCohort Study in Iwate Prefecture

Center or Satellite	Municipality	Population (as of October 1, 2015)	Human damage by the GEJE^a^	Number of participants (as of December 28, 2015)			

				Specific health Table 1checkups	Satellite	Sub total	Total (*n* = 31 838)
Yahaba	Yahaba	27 191	0	790	996	1786	2482
	Tono	27 664	0	0	485	485	
	Ichinoseki	120 379	0	0	211	211	

Kuji	Kuji	35 106	4	2374	455	2829	9953
	Hirono	16 322	0	1440	109	1549	
	Noda	4189	38	423	36	459	
	Fudai	2859	1	287	28	315	
	Ninohe	27 659	0	1858	43	1901	
	Karumai	9275	0	999	18	1017	
	Ichinohe	12 921	0	1270	15	1285	
	Kunohe	5973	0	568	30	598	

Miyako	Miyako	55 017	514	4391	960	5351	8258
	Yamada	15 564	752	1620	25	1645	
	Iwaizumi	9579	7	848	107	955	
	Tanohata	3474	29	286	21	307	

Kamaishi	Kamaishi	35 262	1040	2315	831	3146	4342
	Otsuchi	11 513	1227	1162	34	1196	

Kesen	Ofunato	38 024	419	3256	572	3828	6803
	Rikuzentakata	19 097	1762	2398	64	2462	
	Sumita	5751	0	354	159	513	

GEJE, Great East Japan Earthquake; TMM, Tohoku Medical Megabank Project.

^a^Human damage by the GEJE includes dead and missing persons. The numbers are cited from the General Disaster Prevention Office, Iwate Prefectural Government.

In July 2013, we also started a birth and three-generation cohort study named the TMM Birth and Three-Generation Cohort Study (TMM BirThree Cohort Study) (Table 1 and Figure 5) in one obstetric clinic. The study has since expanded throughout Miyagi Prefecture, and about 50 obstetric clinics and hospitals are now participating in the recruiting process. The main target population of the TMM BirThree Cohort Study is persons living in all municipalities in Miyagi Prefecture (Figure 2). We are recruiting pregnant women at the obstetric clinics and hospitals for the TMM BirThree Cohort, as well as the Community Support Centers in ToMMo.

**Figure 7.**
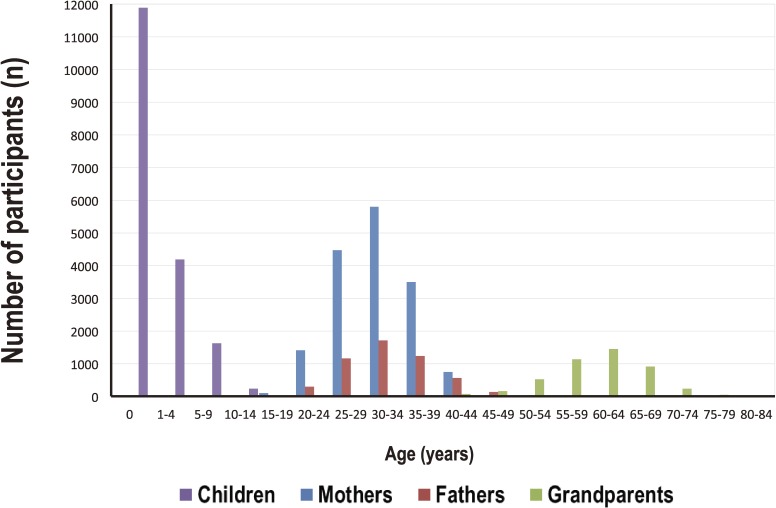
Age distribution of the TMM Birth and Three-Generation Cohort (TMM BirThree Cohort) participants This panel shows the age distribution of children, mothers, fathers, and grandparents who participated in the TMM BirThree Cohort as of December 28, 2015, recruited in Miyagi Prefecture.

We plan to recruit approximately 70 000 participants from three generations for the TMM BirThree Cohort (Table 1). For pregnant women, inclusion criteria are: 1) pregnant women who are registered in the basic resident register of all 35 municipalities in Miyagi Prefecture and 20 municipalities in Iwate Prefecture (Figure 2) at the time of enrollment and whose expected date of delivery is later than February 1, 2014; and 2) pregnant women who visited cooperative obstetric clinics and hospitals in Miyagi Prefecture or one of the seven Community Support Centers in ToMMo. For fetuses and children, inclusion criteria are: 1) fetuses of pregnant women who participated in the study; and 2) siblings of fetuses who participated in the study, regardless of kinship. Finally, for fathers, grandparents, and other family members, inclusion criteria are: father, grandparents, and other family members of fetuses who participated in the study, regardless of kinship. The Exclusion criteria of the TMM BirThree Cohort Study are: 1) persons aged 16 years or over who do not consent to participate in the study; 2) children and fetuses whose legal representatives do not provide consent for them to participate in the study; 3) persons who are not able to fill out study questionnaires, except for children and fetuses; 4) pregnant women who are participating in another birth cohort study for the same pregnancy. As of December 28, 2015, 44 309 persons and 4375 fetuses have already participated in the TMM BirThree Cohort Study (Table 2A). Figure 7 shows the age distribution of 43 658 participants after excluding extended family members. As of December 28, 2015, the participation response rates among pregnant women recruited at obstetric clinics and hospitals for the TMM BirThree Cohort was 67%.

At the time of writing, we have trained more than 200 Genome Medical Research Coordinators (GMRC), who are responsible for informing potential participants about our cohort studies and obtaining consent from them to participate. They are also collecting biospecimens and recording clinical data.

One of the major incentives for participating in the cohort studies is contributing to the implementation of advanced medicine for the next generation. Especially in the TMM BirThree Cohort Study, parents and grandparents seem to have a strong desire to maintain the health of children. Another incentive is receiving the results of blood and urine tests, as well as questionnaire data. We advise all participants to consult their home doctors or go to a hospital if their laboratory or physiological measurements results show any abnormalities. In recompense for their cooperation, we also pay participants 500 to 1000 yen. Additionally, in the TMM BirThree Cohort Study, we send anniversary goods on special occasions, such as a child’s birthday.

We conducted sample size calculation of the cohort studies as follows: In the TMM CommCohort Study, a sample size of 80 000 participants was required for genome-wide screening of gene-by-environment interactions, with minimum odds ratios ranging from 1.4 to 29.1. The sample size was calculated by QUANTO (the Division of Biostatistics, University of Southern California, Los Angeles, CA, USA), which is a program used to calculate the required sample size and power for genetic studies,^30^^,^^31^ under the following assumptions: 1–10% frequency for a risk allele, 10–30% environmental exposure frequency, 90% 5-years follow-up rate, 1–10% disease cumulative incidence, 80% power, and a type I error rate of 4.50 × 10^−10^ for genome-wide context (ie, approximately 1200 × 10^4^ single nucleotide variations (SNVs) and 10 environmental factors). This size would also be sufficient to screen for marginal effects of a few genes or environmental factors.

In sample size calculation in the TMM BirThree Cohort Study, a sample size of approximately 70 000 participants from three generations was required for a multipurpose research platform, including haplotype phasing, parametric linkage analysis, cross-family identity by descent (IBD) mapping,^32^^,^^33^ and family-based association studies, such as the transmission-disequilibrium test (TDT) and the corrected chi-squared test. For example, in the corrected chi-squared test for genome-wide screening of marginal effects at each locus, approximately 20 000 trios gave 80% power to detect variants with odds ratios ranging from 1.4–3.0, assuming: 1–5% frequency for a risk allele, 1% disease prevalence, 80% power, and a type I error rate of 4.00 × 10^−9^ for genome-wide context (ie, approximately 1200 × 10^4^ SNVs). In contrast, approximately 9000 seven-family member groups, all else being equal, would be enough to achieve comparable performance.

There is one more reason to support the calculated sample size for the TMM BirThree Cohort Study. In 2011, the number of annual births in Miyagi Prefecture was 18 062.^34^ If 50% of pregnant women agreed to participate, the number of participants per year would be 9031. Therefore, we set our target of over 20 000 pairs of pregnant women and their fetuses during the recruitment period. We also set targets of 10 000 fathers and 15 000 grandparents (10 000 maternal grandparents and 5000 paternal grandparents), considering the number of pregnant women and feasibility. In 2011, 47% of Japanese pregnant women were primipara.^34^ Thus, we can assume that approximately 50% of fetuses have siblings. We intend to recruit 50% (5000) of these siblings.

For children, we also conducted a cross-sectional study, the ToMMo Child Health Study, every year from 2012 to 2015 in Miyagi Prefecture (Table 1).^35^ This study aimed to investigate and support the health needs of schoolchildren affected by the GEJE. We asked all 35 municipal school boards in Miyagi Prefecture to participate in the study, and 28 of those agreed. The total number of eligible participants was 65 881, and approximately 17 000 (26%) agreed to participate in the study. We distributed the parent-administered questionnaire, which included questions for assessing physical and behavioral status.

The main target diseases of each study are listed in Table 1. We have selected these diseases because of concerns over the potential increase in their prevalence in the affected areas and/or the degree of disease burden on the population in general.

We have assessed many variables in detail. The variables include: 1) a wide range of questions, including sociodemographic factors, lifestyle habits, and medical history; 2) biospecimens’ measurements, including blood, urine, saliva, dental plaque, and breast milk; 3) physiological measurements, including height, weight, body composition, blood pressure or estimated central aortic blood pressure, heart rate, carotid ultrasound imaging, respiratory function, respiratory impedance, calcaneal ultrasound bone mineral density, leg extension strength, grip strength, oral examination, hearing acuity, eye examination, home blood pressure, number of steps per day, waist circumference, visceral fat by bioelectrical impedance analysis, electrocardiogram, pulse wave velocity, and flow mediated dilation; and 4) magnetic resonance imaging (MRI). The assessments, collections, and measurements vary depending on where participants undergo their health examinations. Table 3A and Table 3B show the assessments, collections, and measurements in participants who are 20 years old or over according to the Community Support Center in Miyagi Prefecture and the Satellite in Iwate Prefecture. The assessments, collections, and measurements in participants who are under 20 years old are selected among those in participants who are 20 years old or over considering safety and effectiveness.

**Table 3A.** Assessments, collections, and measurements in participants who are 20 years old or over according to the Community Support Center in Miyagi Prefecture

Questions, biospecimens, or physiological measures	Community Support Center						

	Sendai	Kesennuma	Ishinomaki	Osaki	Tagajo	Iwanuma	Shiroishi
*Questions*							
Questionnaire	+	+	+	+	+	+	+
Touchscreen questionnaire	+	+	+	+	+	+	+

*Biospecimens*							
Blood	C	C	C	C	C	C	C
Urine	+	+	+	+	+	+	+
Saliva	+	+	+	+	+	+	+
Dental plaque	+	+	+	+	+	+	+
Breast milk	M	M	M	M	M	M	M

*Physiological measurements*							
Height/Weight/Body composition	+	+	+	+	+	+	+
Estimated central aortic blood pressure and heart rate	+	+	+	+	+	+	+
Carotid ultrasound imaging	+	+	+	+	+	+	+
Respiratory function	NP	NP	NP	NP	NP	NP	NP
Respiratory impedance	+	+	+	+	+	+	+
Calcaneal ultrasound bone mineral density	+	+	+	+	+	+	+
Leg extension strength	NP	NP	NP	NP	NP	NP	NP
Grip strength	NP	NP	NP	NP	NP	NP	NP
Oral examination	+	+	+	+	+	+	+
Hearing acuity	+	+	+	+	+	+	+
Eye examination							
Intraocular pressure	+	+	+	+	+	+	+
Color retinal photography	+	+	+	+	+	+	+
Axial length	+	+	+	+	+	+	+
Optical coherence tomography	+			+	+	+	
Refraction and keratometry	+						
Home blood pressure	V	V	V	V	V	V	V
Number of steps per day	V	V	V	V	V	V	V
Magnetic Resonance Imaging	VNP						

+ assessed, collected, or measured in all participants who visit the Community Support Center.

^C^ collected in all participants who visit the Community Support Center. Umbilical cord blood is also collected at the time of birth at the obstetric clinics and hospitals.

^M^ collected in participants who are mothers of newborn infants.

^NP^ measured in participants who are not pregnant women.

^V^ measured in participants on a voluntary basis.

^VNP^ measured in participants who are not pregnant women or mothers of newborn infants within 6 months after birth on a voluntary basis.

**Table 3B.** Assessments, collections, and measurements in participants who are 20 years old or over according to the Satellite in Iwate Prefecture

Questions, biospecimens, or physiological measures	Center	Satellite			
	
	Yahaba	Kesen	Kuji	Miyako	Kamaishi
*Questions*					
Questionnaire	+	+	+	+	+

*Biospecimens*					
Blood	+	+	+	+	+
Urine	+	+	+	+	+

*Physiological measurements*					
Height/Weight	+	+	+	+	+
Waist circumference	+	+	+	+	+
Visceral fat by bioelectrical impedance analysis	+	+	+	+	+
Blood pressure and heart rate	+	+	+	+	+
Electrocardiogram	+	+	+	+	+
Pulse wave velocity	+	+	+	+	+
Carotid ultrasound imaging	+	+	+		
Flow mediated dilation	F	F			
Calcaneal ultrasound bone mineral density	+	+	+	+	+
Eye examination					
Color retinal photography	+	+			
Axial length	+	+			
Optical coherence tomography	+	+			

+ assessed, collected, or measured in all participants who visit the Satellite.

^F^ measured in participants who are 50 years old or over.

In the TMM CommCohort Study and the TMM BirThree Cohort Study, we aim to obtain questionnaire data, blood, and urine from all participants. We also collect umbilical cord blood from newborn infants. We have performed physiological measurements of participants who visited the Community Support Centers or the Satellites (Table 3A and Table 3B). MRI is performed on a voluntary basis. As of December 28, 2015, in the TMM CommCohort Study, about 31% of participants in Miyagi Prefecture and 24% of participants in Iwate Prefecture have had physiological measurements, and about 3% of participants in Miyagi Prefecture have undergone MRI examinations. In the TMM BirThree Cohort Study, about 23% of participants have had physiological measurements, and about 2% of participants have undergone MRI examinations. We return the results of the examinations to participants to help improve their health status. If we observe any incidental findings in the laboratory data, except genome analyses, we immediately advise the participants to consult their home doctors or to go to a hospital within a month.

We have also adopted, in part, E-epidemiology,^36^ which aims to collect epidemiological information using electric devices, such as tablet computers and/or mobile phones. We are now partly collecting data using tablet computers and, in the near future, we are planning to collect follow-up data using the Internet.

In order to follow participants for morbidity and mortality, we adopted the following three strategies (Figure 5): 1) questionnaire by mail or use of E-epidemiology; 2) repeated health examinations at the Community Support Centers in Miyagi Prefecture and the Satellites in Iwate Prefecture; and 3) existing data review.

In Miyagi and Iwate Prefectures, morbidity is identified annually using mail or E-epidemiology. We believe that repeated health examinations at the Community Support Centers in Miyagi Prefecture and the Satellites in Iwate Prefecture are essential for follow-up because persons do not always consult physicians whether or not they have some symptoms when they have health problems. Also, repeated health examinations are important because detailed phenotype information, including various continuous and dichotomous variables, might not always be obtained in medical settings. Nevertheless, medical chart review is one of the most important strategies for reviewing existing data. Data in the available population-based cancer registry system can be used to confirm the incidence of cancer among the study participants. Data for death and moving out of the study areas are annually and biannually verified by reviewing the basic resident register in each municipality. Furthermore, data for the medical insurance system and long-term-care insurance system can be used to obtain information regarding morbidity and disability among the study participants.

In Miyagi Prefecture, ToMMo is considering reviewing an electronic health record (EHR) system called the Miyagi Medical and Welfare Information Network (MMWIN).^37^ The MMWIN is designed to unify medical and well-being data by converting traditional medical and health records to electronic files. Furthermore, for infants and schoolchildren, morbidity is also identified by reviewing the records of health check-ups conducted by municipalities and schools. Data in the system for intractable diseases and specific chronic diseases in childhood conducted by municipalities can also be used.

In Iwate Prefecture, IMM is also considering reviewing records of an EHR system when it becomes available. IMM has the methodological advantage of using data from available population-based stroke and heart disease registry systems to confirm the incidence of stroke and heart disease among study participants.

TMM aims to establish domestic and international collaborations between many other genome cohorts and biobanks to achieve the goals of personalized healthcare and medicine. For this purpose, we adopted questionnaires that share more than 90% of their content with other Japanese representative cohort studies, including the Japan Multi-Institutional Collaborative Cohort Study (J-MICC Study)^38^ and the Japan Public Health Center-based Prospective Study (JPHC Study).^39^ Furthermore, we referenced the “PhenX Tool kit”, which aims to create standard measures of environmental exposures to make it easy for investigators to collaborate with each other, when designing our questionnaires.^40^

#### Biobanking

**Figure 8.**
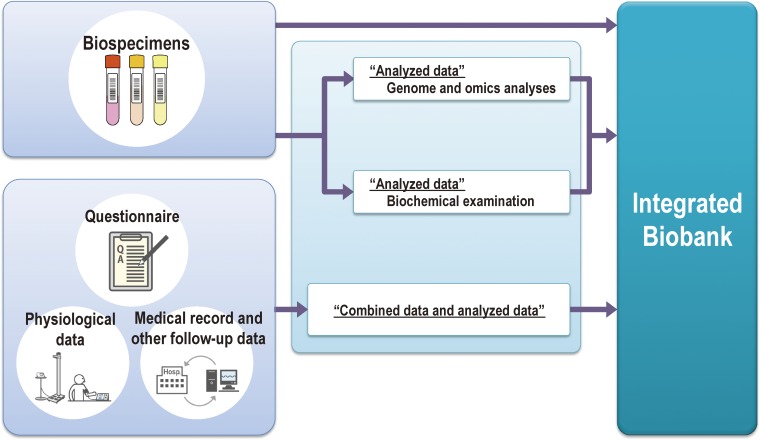
Construction of an integrated biobank We are constructing an integrated biobank, which consists of biospecimens and data of genome and omics linked to de-identified health and clinical information of cohort study participants.

TMM will establish an integrated biobank based on the above-mentioned cohort studies (Figure 8). Because the biospecimens will be depleted over the course of the biobank operation, we decided to conduct routine genome and omics analyses at the ToMMo and IMM research centers and to distribute the information along with the biospecimens. We refer to this idea as the integrated biobank.

**Figure 9.**
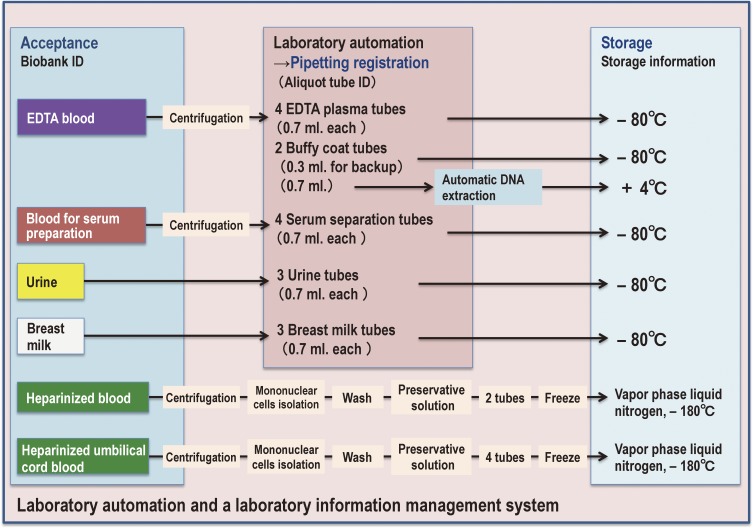
Laboratory automation and a laboratory information management system in TMM This panel shows how TMM handles and stores biospecimens from participants in the TMM BirThree Cohort Study.

The content of biospecimens that we are collecting, as well as the methods for collection and storage, are summarized in Figure 9. As the details of this biobanking process will be reported in a separate paper, here we only briefly describe the issues. One of the important improvements that TMM is adopting is to actively and systemically incorporate laboratory automation and a laboratory information management system to handle and store biospecimens from the participants (Figure 9).

We are planning to facilitate the implementation of personalized healthcare and medicine with well-controlled governance of the biobank management system. The system will transfer the biospecimens and data to the researchers most suited to maximizing their potential. In this way, the biobank will serve as the foundation for developing preventive and treatment methods based on genetic and environmental influences on the onset of diseases.

We will adopt a materials and data transfer policy, which will contribute to the reconstruction of the affected areas. Biobank users are expected to contribute to the conduct of research that targets the potentially increasing diseases in the affected areas and will engage in research that will attract medical industries to these areas.

As one of the representative repositories of biospecimens and data in Japan, we will make every effort to appropriately manage the biobank to meet international standards. The biospecimens and data in our biobank will be shared with many researchers, who may discover mechanisms to explain why some people develop specific diseases while others do not. In addition to genome analysis, TMM will also perform omics analyses, such as transcriptomics, proteomics, and metabolomics, in the prospective cohort settings.

#### Genomic research

Although our biobank aims to serve as the foundation for personalized healthcare and medicine and for developing future preventive and treatment methods by making the biospecimens and data available to the scientific community, we also plan to conduct genomic and omics researches ourselves. The aims of our genomic research are: 1) to construct an integrated biobank as described above; 2) to return genomic research results to the participants of the cohort studies, which will lead to the implementation of personalized healthcare and medicine in the affected areas in the near future; and 3) to contribute to the development of personalized healthcare and medicine worldwide. Our use of any biospecimens and data will be done with permission from the Sample and Data Access Committee of the biobank.

To achieve these aims, the analyzed genomic and omics information will be combined with information derived from the cohort surveys and will be utilized to determine how genetic and environmental factors are linked to diseases. Moreover, the information will be used to create databases for more advanced medicine.

**Figure 10.**
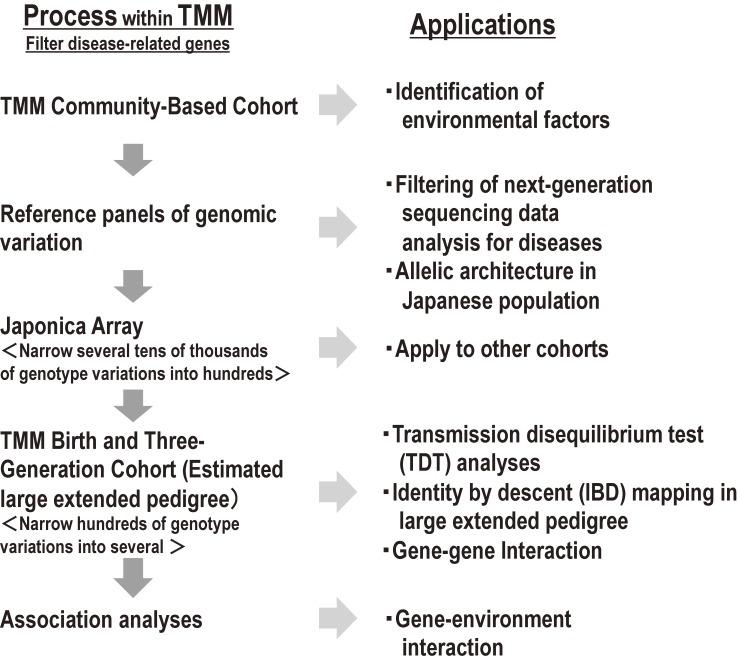
Genome analysis strategies within TMM This panel shows the genome analysis strategies within TMM to realize personalized healthcare and medicine.

We aim to return the results of genome analysis to the participants and to the medical community to implement personalized healthcare and medicine in the near future. To this end, we plan to generate a Japanese reference panel of genomic variation to elucidate the characteristics of the Japanese genome, to open the panel to researchers, and to create a custom-made SNP array (“Japonica Array”)^41^ to detect susceptibility loci to common complex diseases (Figure 10). This strategy will uniquely utilize familial imputation based on the TMM BirThree Cohort, as the usefulness of the realized IBD concept has been recognized^32^^,^^33^ and the power of familial imputation has been demonstrated in the deCODE project.^42^^,^^43^

We have already created one Japanese reference panel of genomic variation, the integrative Japanese Genome Variation Database, which is publicly available online (http://ijgvd.megabank.tohoku.ac.jp/)^44^ and includes allele frequencies of all SNVs. The database is based on the whole-genome sequencing of 1070 samples from the TMM CommCohort, and average sequencing coverage was 32.4×. The database has several distinctive characteristics (Table 4)^44^ and will elucidate DNA sequence variations in the Japanese population.

**Table 4.** Characteristics of a Japanese reference panel of genomic variation, the integrative Japanese Genome Variation Database

Participants	One population of Japanese
Library source	Genomic DNA extracted from peripheral blood cells
Sequencing target	Whole genome
Analysis Setting	One setting at Tohoku University using Illumina HiSeq 2500
Coverage	32.4 × on average

**Figure 11.**
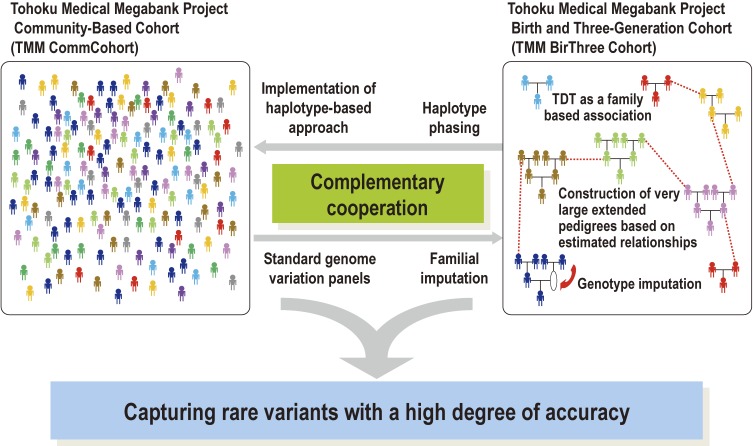
Complementary cooperation of the TMM Community-Based Cohort (TMM CommCohort) and the TMM Birth and Three-Generation Cohort (TMM BirThree Cohort) in genome analyses This panel shows synergetic effects of the TMM CommCohort and the TMM BirThree Cohort.

As mentioned above, the synergetic effects of the TMM CommCohort and the TMM BirThree Cohort will enhance the capture of rare variants with a high degree of accuracy. To achieve this, we will utilize a standard panel of genomic variation based on the next-generation sequencing analysis of the population-based cohort data coupled with familial genotype imputation into the birth and three-generation cohort data (Figure 11).

Omics research is also a big component of our work. We have completed multi-omics (metabolome and proteome) analysis of 500 plasma samples collected from the TMM CommCohort Study. These data have been integrated into the “Japanese Multi Omics Reference Panel (jMorp)” database, which is publicly available online (https://jmorp.megabank.tohoku.ac.jp/). This research may provide biomarker profiles for the early detection of diseases. In our omics study, ToMMo is mainly in charge of proteomics and metabolomics, while IMM is mainly in charge of epigenetics and transcriptomics.

#### Ethical issues

We inform eligible persons of the aims and protocols of TMM and obtained written informed consent from all who agree to participate according to their free will. We adopt general and continuing consent to participate in TMM. At baseline, we explain the protocols applied in the cohort studies, biobanking, and general research methods for genome analyses, omics analyses, and cell culture, which is not intended to differentiate germ-line cells or organ cells. During the follow-up period, we will inform participants of individual research protocols through newsletters, web pages, and/or other communication tools after the researchers have defined the protocol. For the TMM BirThree Cohort Study, participants with parental authority, such as parents or guardians, provide written informed consent for their children to participate in the study.

TMM protocol has been reviewed and approved by the Ethics Committee of Tohoku University Graduate School of Medicine for ToMMo and by the Ethics Committee of Iwate Medical University for IMM. TMM is conducted in accordance with the Declaration of Helsinki,^45^ Ethical Guidelines for Human Genome/Gene Analysis Research,^46^ and all other applicable guidelines.

## DISCUSSION

TMM has two goals: to contribute to reconstruction after the GEJE and to implement personalized healthcare and medicine. To achieve these goals, we have designed the protocols with the following features: 1) compatibility of health improvement and observational study; 2) strategic cohort designs; 3) general and continuing consent; 4) follow-up with an EHR system; 5) an integrated biobank that will contribute to the health of affected persons; and 6) rapid genome analyses following cohort establishment and potential return of genome analysis results.

### Compatibility of health improvement and observational study

A prospective cohort study is an observational epidemiological method that allows limited intervention to improve participants’ health even when researchers find modifiable factors present in the participants. The design aims to observe exposures and subsequent outcomes. Nevertheless, we have decided to intervene when we find modifiable factors at baseline or at follow-up health assessments, since our studies are being conducted in the earthquake- and tsunami-affected areas. Additionally, we immediately advise participants to consult their home doctors or go to a hospital if abnormalities in laboratory or physiological measurements are detected. To appropriately evaluate the intervention effects, we will repeatedly monitor the exposures and outcomes through follow-up every year by mail and every 5 years by reassessments. To monitor health improvements among the participants, both children and adults, we will continuously check official public data to evaluate mortality, morbidity, or disabilities.

### Strategic cohort designs

We have employed two prospective cohort designs. One is a population-based adult cohort study, the TMM CommCohort Study, while the other is a birth and three-generation cohort study, the TMM BirThree Cohort Study.

For the TMM CommCohort study, we have recruited participants mainly from specific health checkup sites, which cover about 76% of the participants in Miyagi Prefecture and about 84% of those in Iwate Prefecture. The study has some limitations. Because those who undergo specific health checkups are more likely to be older and women, the number of young participants and men in our study is relatively small. Our participants might also have a high level of health consciousness, since they attended health checkups. Additionally, our recruitment sites are those that conduct mass examinations and do not include individual medical institutes conducting specific health checkups, so the study population in the recruitment sites might not sufficiently represent the target population (Table 2A and Table 2B). Nevertheless, the TMM CommCohort Study has a large sample size, detailed data (including MRI data), and follow-up with an EHR system.

The TMM BirThree Cohort Study is, to our knowledge, the first of its kind in the world. Family members have been traditionally included in research that investigates single-gene diseases. However, we developed a three-generation design because we believe that this could potentially have the power to detect clues to explain gene-environment related diseases. As has been discussed by Stolk et al, a three-generation design has the advantages of finding *de novo* variation and directing haplotype assessment.^47^ The potential importance of the use of a three-generation design is also suggested by a study showing that advanced grandparental age was associated with increased risk of autism.^48^

To our knowledge, the only relatively large-scale population-based cohort study with a three-generation design is the “LifeLines Cohort Study (LifeLines)” in the Netherlands.^47^^,^^49^ LifeLines has many potential advantages for personalized healthcare and medicine, because sufficient family information is collected. One salient difference between LifeLines and TMM is that LifeLines recruited children but not fetuses.^49^ Childhood recruitment may not allow sufficient investigation of *in utero* exposures. The Developmental Origins of Health and Disease (DOHaD) hypothesis indicates that the fetal environments may strongly influence subsequent health in childhood, adolescence, and adulthood.^50^^–^^52^ Our TMM BirThree Cohort Study design is a prospective cohort from early fetal life onward and could therefore allow us to decipher interactions between genes and lifetime environmental factors.

For children, we also conducted the ToMMo Child Health Study in Miyagi Prefecture for school-aged children, in addition to the two cohort studies (Table 1).^35^ Through our two prospective cohort studies and one cross-sectional study, we are able to assess the health status of residents of all ages, from fetuses to elderly persons. Including all ages is necessary because persons of all ages have been affected by the GEJE and will demand advanced medicine for potentially increasing diseases in the near future.

Combining population-based and family-based cohort studies like TMM is quite efficient in elucidating interactions between genetic and environmental factors and in deciphering the etiology of common complex diseases, which are derived from gene-environment interactions and affected by rare variants (Figure 10 and Figure 11).^53^ Indeed, Styrkarsdottir et al identified four new genome-wide significant loci harboring a rare predisposing variant in a study of bone mineral density using methods similar to those mentioned above for the deCODE project.^54^

Our study sites include not only severely affected coastal areas but also inland areas in Miyagi and Iwate Prefectures to allow for adjustment of the effects of the GEJE on the cohorts. Furthermore, to validate our results and to obtain sufficient statistical power, we will compare the results of our cohort studies with those conducted in other areas of Japan, which include both prospective and case-control designs.^38^^,^^39^^,^^55^^–^^60^

### General and continuing consent

In the biobanking project, sufficient informed consent at the time of study enrollment is, in general, believed to be practically impossible.^61^ To maximize participants’ hopes for personalized healthcare and medicine, we adopted general and continuing consent to participate in the TMM cohort studies.

We will continuously provide detailed protocol information of individual research methods once the protocols have been determined. Also, we plan to contact the participants once a year, at which time we will obtain, if necessary, re-consent to participate in TMM according to the progression of the study.

### Follow-up with an EHR system

Data from the integrated EHR system will be gradually used as a data source to follow participants in our cohorts.^62^^,^^63^ The EHR generally includes laboratory testing results, disease diagnoses, and treatment information, including use of medications. To our knowledge, there has been no cohort study with follow-up of participants linked to EHR in Japan.

### Integrated biobank

The integrated biobank of TMM consists of biospecimens and genome and omics data linked to de-identified health and clinical information of the cohort studies’ participants. We aim to establish the TMM biobank to maximize the potential of the biospecimens and data and to contribute to realize advanced personalized healthcare and medicine.

The TMM biobank has a unique policy of transferring biospecimens to researchers who can aid persons affected by the GEJE, because the biobank’s primary aim is to reconstruct the affected areas.

### Rapid genome analyses and potential return of genomic research results

Genomic research in TMM includes construction of an integrated biobank and potential return of genome analysis results to implement personalized healthcare and medicine. There are at least a few population studies that have revealed genomic information to participants. One example is a Finnish cohort study that returned information regarding long QT syndrome genes.^64^ Although many barriers must first be addressed before the return of genome analysis results to participants, we have started to consider ethical, legal, and social issues of returning such information and implementing personalized healthcare and medicine.

### Conclusion

We have launched a novel project TMM to recover from the GEJE and to address diseases related to the disaster, including the TMM strategic cohort studies, biobanking, and genomic research. TMM will serve present and future generations by providing personalized healthcare and medicine.

## ONLINE ONLY MATERIAL

eAppendix 1. The Tohoku Medical Megabank Project Study Group members.

Abstract in Japanese.
