# Supplementary material for: The Tohoku Medical Megabank Project: Design and Mission
Source: J Epidemiol. 2016 Sep 5;26(9):493–511. doi: 10.2188/jea.JE20150268 (PMC5008970; doi:10.2188/jea.JE20150268)
Supplement: eAppendix 1. [file je-26-493-s001.pdf]

eAppendix 1. Tohoku Medical Megabank Project (TMM) Study Group

The Tohoku Medical Megabank Project Study Group members as of December 1, 2015 are:

Tohoku University Tohoku Medical Megabank Organization (ToMMo)**,** ***Principal Investigator:*** Masayuki Yamamoto. ***Study Group Members:*** Michiaki Abe, Mitsunari Abe, Tomohiko Arai, Koichi Chida, Inaho Danjoh, Masayuki Ebina, Shinichi Egawa, Nobuo Fuse, Takanori Hasegawa, Hiroaki Hashizume, Waku Hatta, Toshio Hattori, Masahiro Hiratsuka, Kei Homma, Atsushi Hozawa, Kazuhiko Igarashi, Noriko Ishida, Naoto Ishii, Tadashi Ishii, Mami Ishikuro, Kiyoshi Ito, Sadayoshi Ito, Yoshitsugu Iwakura, Maiko Kageyama, Hiroshi Kaneko, Yasutake Katoh, Fumiki Katsuoka, Yoshio Kawaguchi, Yosuke Kawai, Hiroshi Kawame, Junko Kawashima, Masahiro Kikuya, Osamu Kimura, Tomoya Kimura, Kengo Kinoshita, Kazuyuki Kitatani, Hideyasu Kiyomoto, Tomoko Kobayashi, Kaname Kojima, Yutaka Kondo, Seizo Koshiba, Hisaaki Kudo, Shigeo Kure, Miho Kuriki, Shinichi Kuriyama, Yoko Kuroki, Mitsuyo Matsumoto, Hirohito Metoki, Takahiro Mimori, Naoko Minegishi, Kazuharu Misawa, Eikan Mishima, Masako Miyashita, Satoshi Mizuno, Ikuko N. Motoike, Satoshi Nagaie, Fuji Nagami, Masao Nagasaki, Sachiko Nagase, Yu Nakagome, Naoki Nakamura, Tomohiro Nakamura, Junichi Nakata, Jun Nakaya, Naoki Nakaya,

Keiko Nakayama, Naoki Nariai, Yoko Narikawa, Akira Narita, Ichiko Nishijima, Kensuke Nishimiya, Takahiro Nobukuni, Taku Obara, Soichi Ogishima, Noriaki Ohuchi, Yoshikiyo Ono, Noriko Osumi, Hiroshi Otsu, Xiaoqing Pan, Daisuke Saigusa, Rumiko Saito, Sakae Saito, Masaki Sakaida, Mika Sakurai-Yageta, Emiko Sato, Yuki Sato, Yukuto Sato, Atsushi Sekiguchi, Tomoko F. Shibata, Osamu Shimizu, Ritsuko Shimizu, Kazuro Shimokawa, Toru Shimosegawa, Naru Shiraishi, Matsuyuki Shirota, Junichi Sugawara, Kichiya Suzuki, Yoichi Suzuki, Takako Takai-Igarashi, Yasuyuki Taki, Gen Tamiya, Osamu Tanabe, Hiroshi Tanaka, Yukari Tanaka, Takahiro Terakawa, Jin Teshima, Soichiro Toda, Teiji Tominaga, Hiroaki Tomita, Akito Tsuboi, Naho Tsuchiya, Kaoru Tsuda, Ichiro Tsuji, Masao Ueki, Ryu Watanabe, Nobuo Yaegashi, Junya Yamagishi, Yumi Yamaguchi-Kabata, Chizuru Yamanaka, Riu Yamashita, Nobuhiro Yaoita, Jun Yasuda, and Junji Yokozawa.

Iwate Medical University Iwate Tohoku Medical Megabank Organization (IMM), Principal Investigator: Kenji Sobue. ***Study Group Members:*** Ryujin Endo, Akimune Fukushima, Ryohei Furukawa, Tsuyoshi Hachiya, Jiro Hitomi, Takeo Kasai, Seiichiro Kobayashi, Motoyuki Nakamura, Satoshi Nishizuka, Kuniaki Ogasawara, Hideki Ohmomo, Kotaro Otsuka, Kotaro Oyama, Akio Sakai, Kiyomi Sakata, Makoto Sasaki, Ryohei Sasaki, Mamoru Satoh, Atsushi Shimizu, Yuh Shiwa, Nobuyuki Takanashi, Kozo Tanno, and Kayono Yamamoto.
