# Supplementary material for: The Tohoku Medical Megabank Project: Design and Mission
Source: J Epidemiol. 2016 Sep 5;26(9):493–511. doi: 10.2188/jea.JE20150268 (PMC5008970; doi:10.2188/jea.JE20150268)
Supplement: Abstract in Japanese. [file je-26-493-s002.pdf]

The Tohoku Medical Megabank Project: Design and Mission

東北メディカル・メガバンク計画：デザインとミッション

栗山進一[1-3*]，八重樫伸生[1,2,4]，長神風二[1]，新井知彦[1]，川口悦生[1]，大隅典子[1,2]，境田正樹[1]，鈴木洋一[1,2]，中山啓子[1,2]，橋詰拓明[1]，田宮元[1]，川目裕[1,2]，鈴木吉也[1,2,4]，寳澤篤[1,2]，中谷直樹[1,2]，菊谷昌浩[1,2]，目時弘仁[1,2]，辻一郎[1,2]，布施昇男[1,2,4], 清元秀泰[1,2,4]，菅原準一[1,2,4]，坪井明人[1,4,5]，江川新一[1-4]，伊藤潔[1-4]，千田浩一[1-4]，石井正[1,2,4]，富田博秋[1-4]，瀧靖之[1,2,6]，峯岸直子[1,2]，石井直人[1,2]，安田純[1,2]，五十嵐和彦[1,2]，清水律子[1,2]，長﨑正朗[1]，小柴生造[1]，木下賢吾[1,7]，荻島創一[1]，高井貴子[1]，冨永悌二[1,2,4]，田邉修[1,2]，大内憲明[1,2,4]，下瀬川徹[1,2,4]，呉繁夫[1,2,4]，田中博[1]，伊藤貞嘉[1,2,4]，人見次郎[8,9]，丹野高三[8,9]，中村元行[8,9]，小笠原邦昭[8,9]，小林誠一郎[8,9]，坂田清美[8,9]，佐藤衛[8,9,10]，清水厚志[8]，佐々木真理[8,9,10]，遠藤龍人[8,9]，祖父江憲治[8,9,10]，東北メディカル・メガバンク計画スタディ・グループ*，山本雅之[1,2]

[1] 東北大学　東北メディカル・メガバンク機構, [2] 東北大学　大学院医学系研究科, [3] 東北大学　災害科学国際研究所, [4] 東北大学病院, [5] 東北大学　大学院歯学研究科, [6] 東北大学　加齢医学研究所, [7] 東北大学　大学院情報科学研究科, [8] 岩手医科大学　いわて東北メディカル・メガバンク機構, [9] 岩手医科大学　医学部, [10] 岩手医科大学　医歯薬総合研究所

2011 年 3 月 11 日に発生した東日本大震災は、東北地方の太平洋沿岸に甚大な被害をもたらした。震災からの復興を促進し、災害後の医療上の問題を解決するために、東北大学は東北メディカル・メガバンク計画を立案し、岩手医科大学と協働して、宮城県と岩手県において、地域住民コホート調査及び三世代コホート調査と名付けた 2 つの前向きコホート調査を開始した。地域住民コホート調査では、被災地を中心に 80,000 人の成人をリクルートする、また、三世代コホート調査では、妊婦と配偶者、出生した子供、その兄姉、祖父母あわせて 70,000 人をリクルートする予定である。前者では、2013 年から 2016 年まで参加者のリクルートを実施し、その後少なくとも 5 年間追跡する、また、後者では、2013 年から 2017 年まで参加者をリクルートし、その後少なくとも 4 年間追跡する予定である。これらに加え、2012 年から宮城県で横断調査デザインからなる地域子ども長期健康調査を開始している。東北メディカル・メガバンク計画では、これら 2 つの前向きコホート調査をもとに、生体試料とゲノム情報を含む関連情報から構成される複合バイオバンクを構築する。本複合バイオバンクには、健康と医療に関する情報、生体試料、ゲノムデータ等が保管され、主として東日本大震災によって増加や症状の悪化等が懸念される疾患について個別化予防・医療を実現するための基盤を確立することを目指している。なお、生体試料や情報は、外部の研究者へも分譲する予定である。本計画では、これらの活動を通して東日本大震災の住民の健康への中長期的な影響を調査する。東北メディカル・メガバンク計画では、コホート調査、バ

イオバンク形成とともに、ゲノム解析研究を推進する。その目的は、1）複合バイオバンクを構築する、2）コホート調査の参加者へゲノム解析結果を回付し、被災地において個別化予防・医療を推進する、3）人類全体に対して、個別化予防・医療の発展に貢献する、ことである。東北メディカル・メガバンク計画の活動を通して、大規模災害で被害を受けた地域における医療問題の解決方法及び疾病予防のためのゲノム情報の有益さを明らかにすることを目指す。

＊東北メディカル・メガバンク計画スタディ・グループの名簿は Appendix を参照

キーワード：東日本大震災；東北メディカル・メガバンク計画；コホート調査；バイオバンク；ゲノム解析研究
